# The Need for Precision Therapies as Determined by Genetic Signature for Cystic Fibrosis

**DOI:** 10.3390/jpm11121353

**Published:** 2021-12-12

**Authors:** Farah R. Zahir

**Affiliations:** Department of Medical Genetics, University of British Columbia, Vancouver, BC V6H 3N1, Canada; farahz@bcchr.ca

Cystic fibrosis (CF) is a devastating genetic infant-onset disease. Caused by autosomal recessive mutations in the *CFTR* gene, individuals who are homozygous or compound heterozygous for damaging variants present a variety of symptoms affecting multiple systems, with respiratory infections being the most frequent presentation [1,2]. Characteristic of this disease is the producing of thick mucus in the lungs that can lead to the patient ‘choking’. Other respiratory disease symptoms include frequent lung infections and inflammation; this symptom class constitutes the major cause of death for CF patients [1,2]. In the not-so-distant past, children born with this ‘double hit’ had a very low life expectancy. However, this changed once the major gene causing CF, the CF transmembrane conductance regulator (*CFTR*) gene, was discovered in 1989 [3] and newborn screening was established in most countries to diagnose the condition early [4].

As research in *CFTR* (and the protein the gene produced, also termed CFTR) expanded, so did our understanding of the gene’s function, the outcome of various mutations in the gene, and their effect on multiple organ systems. CFTR forms a molecular channel controlling ion concentration in cellular and glandular secretions of the respiratory, GI, reproductory tracts, and sweat glands. Currently over 2000 pathogenic variants are recognized in *CFTR* [2]. Many of the variants display distinct population/ethnic profiling [4,5,6,7,8]. The F508del mutation, for example, has been reported to be present in at least one allele for 82.5% of European patients [2]. However, we note that while mutations in *CFTR* have been extensively studied in Caucasian CF patients, studies profiling other ethnicities are not as comprehensive [7]. Conjointly, development of new treatments and therapies are almost inordinately skewed toward mutations predominant in Caucasian populations.

With the advent of the ‘omics era, precision medicine initiatives are further increasing our understanding of genetic causes for CF and possible precision therapies (as evidenced by this paper by Laselva et al. [9]). Small molecular modular therapy for CF developed during the past decade has led to dramatic improvements in treatment and quality of life for patients [10]. They include pharmacological agents that are able to target specific defects in CFTR, categorized as potentiators, correctors, and so on {see [10] for a review}. However only a few *CFTR* mutations are currently targeted by FDA approved small molecule modular therapies. Specifically, the currently approved therapies are focused to *CFTR* mutations highly prevalent in North American populations as explained above.

In this paper, the authors, though based primarily in Canada, make an admirable effort to investigate a small molecule modular therapy combination that can be effective against variants frequent in non-Caucasian ethnicities. They report studies on the c.3700A>G variant that is highly prevalent in Middle Eastern populations, reported as its second most common [11,12,13], and the W1282X mutation found in Jewish populations. They build upon their and other’s previous efforts to characterize the effect of c.3700A>G, which affects nucleotide binding domain-2 of the CFTR protein. They examine the effect of several combinations of trademarked as well as experimental modulators on various cellular models, notably also including derived nasal epithelial cultures from patients homozygous for the c.3700A>G mutation (as well as those heterozygous for it (c.3700A>G/W1282X)). Their results, though preliminary, show a promising rescue of CFTR protein to functional levels using the modulator combination AC1 + AC2-2 + AP2. Though the authors do not discuss why this particular combination proved more effective than others in any great depth, and they do report varying results across the several cellular models they investigated. Nevertheless, the results are striking and warrant rigorous and extensive follow up studies.

A notable feature of this study is the excellent example it provides of several scientists and groups in many different centers co-operatively working together. The authors note many cell models they used as being gifts from other researchers, and frequently cite the works of others in the area. This is refreshing indeed, as too often modern-day scientists miss opportunities to make impactful discoveries due to a culture that can be often focused on self-success rather than finding the cure for the disease. Here we see enhanced results due to a research community sharing vital cell model resources, enabling a more rigorous testing for novel/effective modulator combinations.

Finally, this study (as with others [14,15,16]) is showing how highly precise genetic knowledge is leading to formulating of precision therapies. Indeed, the continuously unfolding picture of CF and the gene mutations that cause it, calls for precisely such an approach to find more and better treatments.
